# Risk factors for cholera mortality: A scoping review

**DOI:** 10.1111/tmi.14106

**Published:** 2025-04-02

**Authors:** Despina Pampaka, Kathryn Alberti, David Olson, Iza Ciglenecki, Philippe Barboza

**Affiliations:** ^1^ Global Task Force on Cholera Control Geneva Switzerland; ^2^ Global Task Force on Cholera Control Secretariat Geneva Switzerland; ^3^ Médecins Sans Frontières Geneva Switzerland

**Keywords:** case fatality, cholera, cholera mortality, risk factors, scoping review, *vibrio cholerae*

## Abstract

**Objectives:**

Cholera is an easily treatable disease, but many people are still unnecessarily dying from it. To improve current case management practices and prevent mortality requires a comprehensive understanding of who is at higher risk of dying. To identify the most common risk factors, a scoping review was undertaken, to explore the literature and summarise the evidence on cholera mortality and reported risk factors.

**Methods:**

Following the scoping review framework proposed by Arksey and O'Malley (2005), Pubmed, EMBASE, Web of Science, LILACS, Scielo, Cochrane and Open Grey and African Journals Online were searched on 24 November 2021, without restrictions in language or date. After screening and assessing the records across predefined criteria, we performed a thematic analysis on mortality.

**Results:**

A total of 77 studies were included in the final review. The potential reasons explaining the observed mortality were classified in the following categories: Patient characteristics; Healthcare; and Health‐seeking behaviour. The identified risk factors were multi‐dimensional, inter‐dependent and context‐specific. When exploring the patients' characteristics, the available data suggested that in many contexts, case fatality ratios were higher among males and older people, especially those aged 50 or above. Twelve studies reported the place of death, with the percentage of community deaths ranging from 23% to 96%. Evidence on comorbidities and cholera deaths was too scarce for analysis.

**Conclusions:**

Cholera has been a disease of global importance for more than two centuries. Despite this, our review highlighted that there has been limited published evidence about factors that increase the risk of cholera‐related death. Collecting, reporting and analysing baseline characteristics such as age, sex and predisposing conditions can improve our understanding of cholera mortality risk factors and guide improvements in future case management recommendations.

## INTRODUCTION

Cholera is a water‐borne disease caused by the bacterium *Vibrio cholerae*, serogroups O1 and O139. It is characterised by acute diarrhoea and dehydration, which can be severe and fatal if left untreated. Cholera is epidemic‐prone and remains a major global public health threat affecting populations experiencing the effects of a lack of economic development, conflict and climate change. The burden of disease is considerable, with an estimated 2.9 million cases and 95,000 deaths annually [1]. Cholera can be prevented by ensuring access to clean water and adequate sanitation.

Severe cholera leading to death is a result of hypovolemic shock and critical organ underperfusion and failure. Fortunately, cholera‐related deaths can be almost entirely prevented by early detection of cases, rapid access to care, and adequate treatment focussed on rehydration. One of the two overall objectives of the Global Task Force on Cholera Control (GTFCC) Ending Cholera: a global roadmap to 2030 is to reduce cholera deaths by 90% [2]. While progress has been made with the availability of oral rehydration solution (ORS) and medical workers able to give intravenous (IV) fluid replacement, the recommended case fatality ratio threshold (CFR) of <1% is often exceeded [3], showing that further actions must be taken.

Current cholera case management protocols focus on standard rehydration with adaptation for specific groups known to be at higher risk of dying while treated at health facilities. These include children with severe acute malnutrition and pregnant women in whom the risk is related to foetal mortality [4, 5, 6]. Experience from those working in cholera‐affected settings suggests that there are further groups at risk of dying from cholera, such as older patients, young children and patients with comorbidities. The elderly, with an increasing likelihood of cardiovascular disease, may not be able to tolerate the hypovolemic shock, which may make them more likely to die than healthy young adults. At the other end of the age spectrum, young children may not have the mature organ function that permits, at least temporarily, adequate fluid compensation in the face of severe fluid loss due to cholera. Co‐existing infections may also contribute to cholera mortality. For instance, advanced HIV could reduce any immune protection that may occur naturally from previous cholera infection or from cholera vaccination, though this is unproven.

Understanding who is at greater risk of dying from cholera and what other factors contribute to cholera‐related mortality is essential for adapting case management practices, addressing avoidable mortality in otherwise treatable cases. With this in mind, we conducted a scoping review to explore the published evidence to identify risk factors for cholera mortality.

## METHODS

### Study design

We conducted a scoping review using the framework proposed by Arksey and O'Malley [7]. A scoping review was preferred over other types of review as it focuses on the breadth of available information and permits the identification of research gaps. Results were reported following the Preferred Reporting Items for Systematic Reviews and Meta‐Analyses (PRISMA) Extension for Scoping Reviews (PRISMA‐ScR) guidelines (Table S1).

### Research questions

Two research questions were formulated: (1) What has been described about cholera mortality, both in community and health facilities? (2) What are the reported risk factors for cholera mortality?

### Search strategy

An extensive search of the databases Pubmed, EMBASE, Web of Science, LILACS, Scielo, Cochrane and Open Grey and of the journal African Journals Online, was conducted on 24 November 2021, without restrictions in language or date. The key words and Medical Subject Headings MeSH terms used were “cholera”, “mortality”, “death”, “fatal outcome”, “fatal” and “case fatality ratio” combined using Boolean operators. The full search strategy is provided in Table S2.

### Eligibility criteria

We predefined a list of eligibility criteria in order to select the studies for the review. We excluded studies that referred to periods prior to the 7th cholera pandemic, that is, before 1961. The inclusion criteria included: (i) studies on human populations; (ii) infections with *Vibrio cholerae* (O1 or O139); (iii) at least two reported fatal cholera cases; and (iv) descriptive characteristics of fatal cases.

### Study selection

The retrieved records were uploaded on the application Rayyan QCRI for Systematic Reviews, and duplicates were deleted. A three‐step process was applied to screen the records. First, titles and/or abstracts of the records were screened to identify papers relevant to the research question. Second, the full text of the relevant papers was reviewed, and those not meeting the eligibility criteria were excluded. Finally, backward snowballing, that is, manual search of reference lists to identify further studies missed by the electronic database search, was conducted.

### Data extraction

A data collection form was prepared to guide the data charting (data extraction). The included records were reviewed to extract details describing the studies, participants, decedents, case fatality ratio (CFR) and any key information relevant to the research questions. In addition, we extracted findings or comments about the mortality and/or the CFR observed in the study. These factors were not restricted to quantitative information or statistical measures provided in the studies; many of them were extracted from comments and interpretations provided by the authors in the discussion.

### Data analysis

The main characteristics of the included studies were presented in tables as frequencies and percentages. A map of the included studies was produced using Datawrapper. In addition, we summarised the information on the location of death. The findings and comments about deaths and/or CFR and potential risk factors were collated, and a thematic analysis was performed based on the identified factors.

The frequency of studies that reported CFR per age group and per sex was calculated. If a study did not report CFR per age group or per sex, available charted data were used to compute these values; the number of deaths in a specific category was divided by the total number of cases in the same category.

Finally, the analysis was narrowed down to the studies that calculated cholera mortality, examined characteristics of cholera‐related deaths and assessed or presented hypotheses for risk factors.

## RESULTS

### Study selection

**FIGURE 1:**
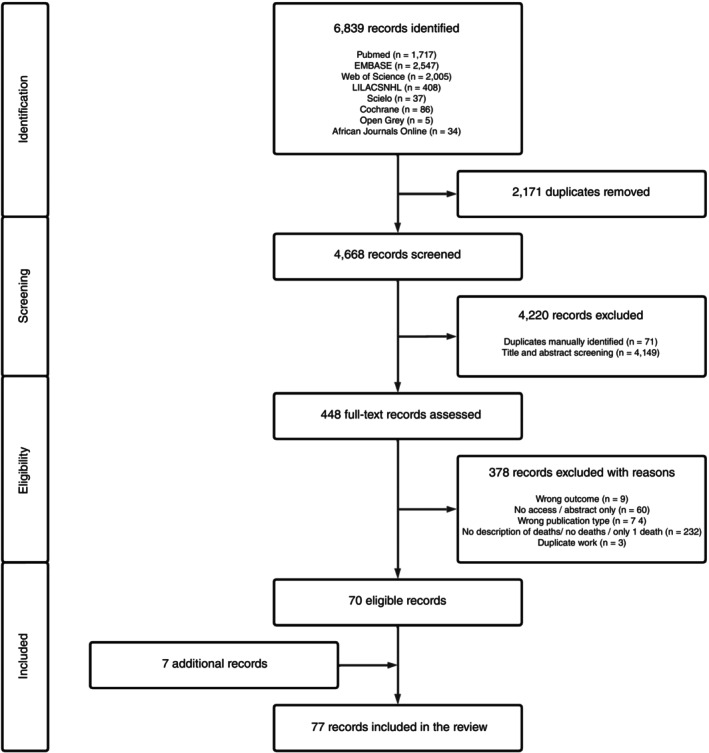
Reporting Items for Systematic Reviews and Meta‐Analyses (PRISMA) flow diagram of study selection.

The database search yielded 6839 documents, of which 2171 were duplicates that were removed. Another 4220 records were excluded after title and/or abstract screening and 378 during the full‐text assessment (Figure 1); leaving 70 records eligible for the review. We identified a further seven studies through snowballing, thus the total number of articles described in this review was 77 [8, 9, 10, 11, 12, 13, 14, 15, 16, 17, 18, 19, 20, 21, 22, 23, 24, 25, 26, 27, 28, 29, 30, 31, 32, 33, 34, 35, 36, 37, 38, 39, 40, 41, 42, 43, 44, 45, 46, 47, 48, 49, 50, 51, 52, 53, 54, 55, 56, 57, 58, 59, 60, 61, 62, 63, 64, 65, 66, 67, 68, 69, 70, 71, 72, 73, 74, 75, 76, 77, 78, 79, 80, 81, 82, 83, 84].

### Main characteristics of the included studies

**FIGURE 2:**
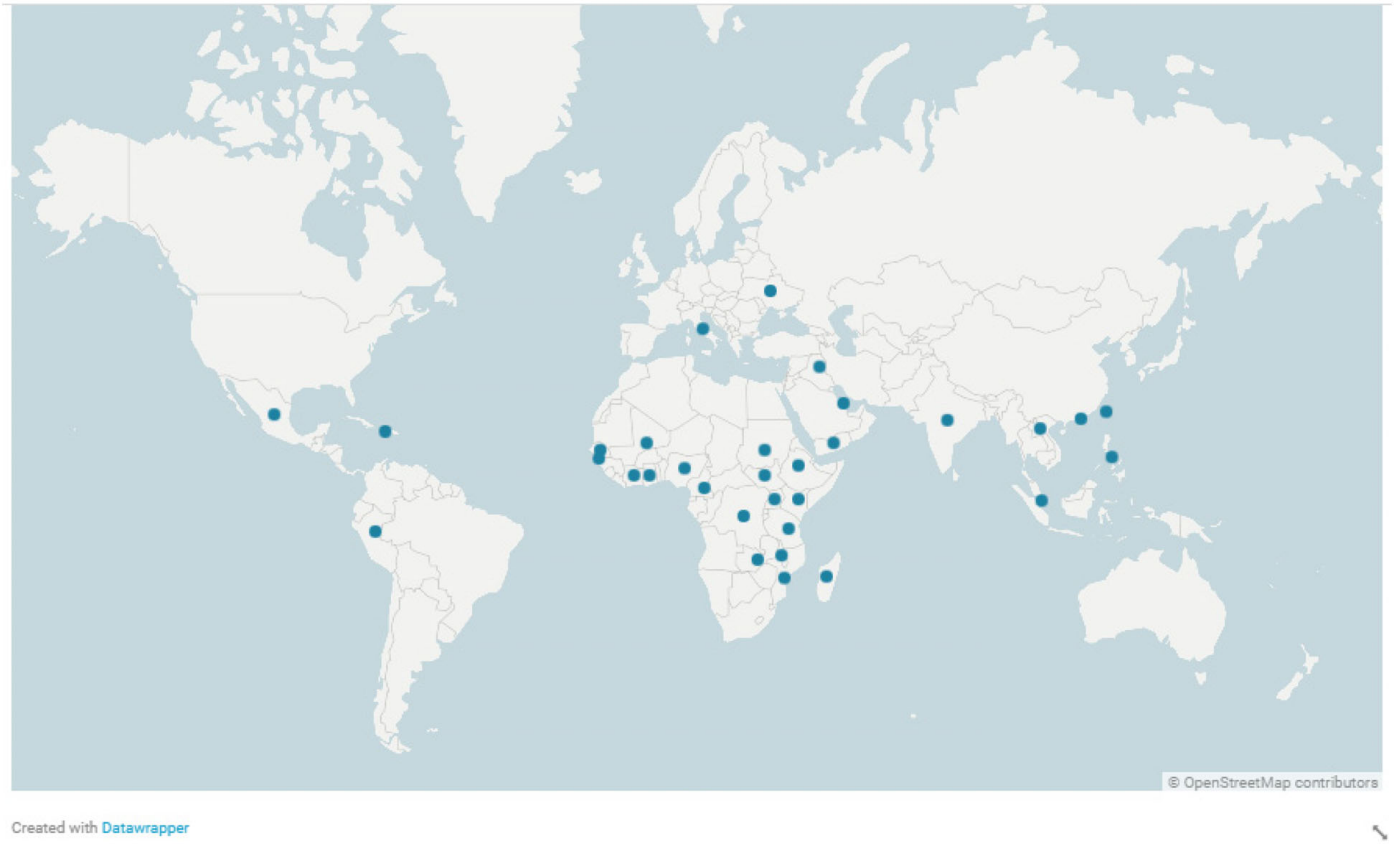
Geographic distribution of studies included in the risk factors for cholera mortality scoping review (*n* = 77). Each dot represents a country and may correspond to more than one study.

**FIGURE 3:**
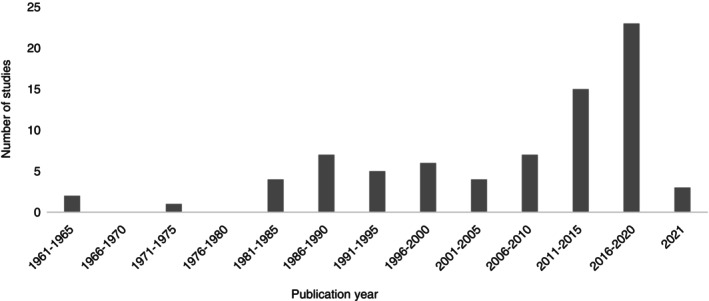
Publication period of the studies included in the risk factors for cholera mortality scoping review (*n* = 77).

The geographic distribution of the included studies is illustrated in Figure 2. Forty‐nine (63.6%) studies were from countries in the African region. Two studies described the cholera outbreak in Lusaka, Zambia in 2017–2018 but were both included as they had a study overlap of only 3 days [56, 71]. The publication year ranged from 1963 [84] to 2021 [32] with an increasing trend in publications over time (Figure 3), with 38 articles (49.4%) published in the decade 2011–2020.

Studies were in both epidemic and endemic settings (Table 1). The number of cases varied from 50 in a small outbreak in Kaduna State, Nigeria [42] to 1,103,683 during a three‐year period in Yemen [21]. Eleven studies reported fewer than 10 deaths [10, 25, 29, 39, 42, 44, 50, 66, 74, 82, 84], while the maximum number of deaths reported was 7436 during the first two years of the epidemic in Haiti that began in 2010 [15].

**TABLE 1:** Main characteristics of the included studies (*n* = 77).

Characteristic	*N*	(%)
Type		
Conference abstract	2	(2.6)
Peer‐reviewed paper	72	(93.5)
Report	2	(2.6)
Review	1	(1.3)
Context of transmission		
Epidemic	43	(55.8)
Endemic	4	(5.2)
Epidemic and endemic/inter‐epidemic periods	29	(37.7)
Unspecified	1	(1.3)
Study design		
Descriptive	39	(51.3)
Descriptive and analytical	38	(48.7)
Case fatality ratio reported or calculated		
Yes	67	(87.0)
No	10	(13.0)
Mortality assessment or death investigation was one of the objectives of the study		
Yes	27	(35.1)
No	50	(64.9)

Three in four studies (*n* = 59, 76.6%) mentioned culture techniques or the use of rapid diagnostic tests to confirm aetiology. Focusing on the studies that provided microbiological information (*n* = 53), *Vibrio cholerae O1* was reported in all of the studies (*n* = 53) and *Vibrio cholerae O139* in three (*n* = 3). El Tor (*n* = 28) and Ogawa (*n* = 24) were the most commonly reported biotypes and serotypes, respectively.

### Case fatality ratio

Sixty‐eight studies reported the overall CFR (not restricted to facility) or provided the total number of deaths and cases (Table 1). The CFR ranged from 0.09% during the first wave of the 2016–2017 cholera outbreak in Hodeidah City, in Yemen [10] to 29% during the 1984 epidemic in Mali in an area with concurrent famine [77]. Thirty‐eight studies (49.4%) used statistical methods overall, of which 28 employed regression analysis or tests of significance specifically for mortality.

### Facility versus community deaths

**FIGURE 4:**
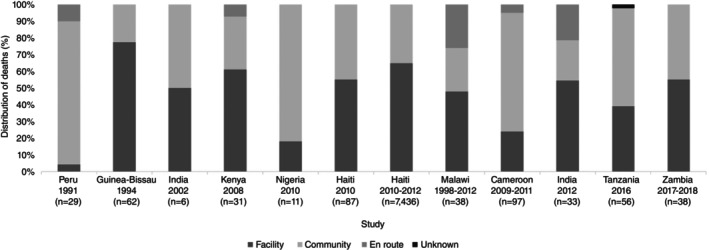
Location distribution of death occurrence—facility versus community (*n* = 12).

The location of deaths was described in 12 studies [12, 15, 40, 53, 55, 56, 59, 62, 63, 65, 66, 69]. Deaths occurred in facilities (hospitals, cholera treatment centres) or in the community (at home, en route to the facility). Figure 4 depicts the distribution of deaths; some studies specified the percentage of the en route deaths, while others used only the community classification. The highest percentage of community deaths (96%) was observed in a study from Peru during the 1991 epidemic [62]. In the remaining studies, the overall percentage of community deaths (including en route) ranged from 23% to 82%. There was limited information about the characteristics of decedents stratified by place of death. Routh and colleagues undertook a rapid mortality assessment during the Haiti 2010 epidemic and compared facility and community decedents [65]. Their findings showed that more facility decedents had used ORS before seeking care (*n* = 23/48, 48%) than community decedents (*n* = 9/39, 23%), with the hypothesis that at the beginning of the cholera epidemic, when this study was conducted, there was insufficient knowledge and use of ORS. Proxies of 81% (*n* = 30/39) community decedents and 69% (*n* = 33/48) health facility decedents reported receiving information about cholera after the outbreak started.

### Risk factors for cholera mortality

The characteristics of patients who died or hypotheses presented by authors explaining the mortality or CFR observed in the included studies were grouped into three main categories (Table S3) that is patient (demographic characteristics, comorbidities), healthcare (access to care, case management, facilities), and health‐seeking behaviour. These categories and sub‐categories were not defined a priori but emerged after data collation.

#### Patient characteristics

##### Sex

**FIGURE 5:**
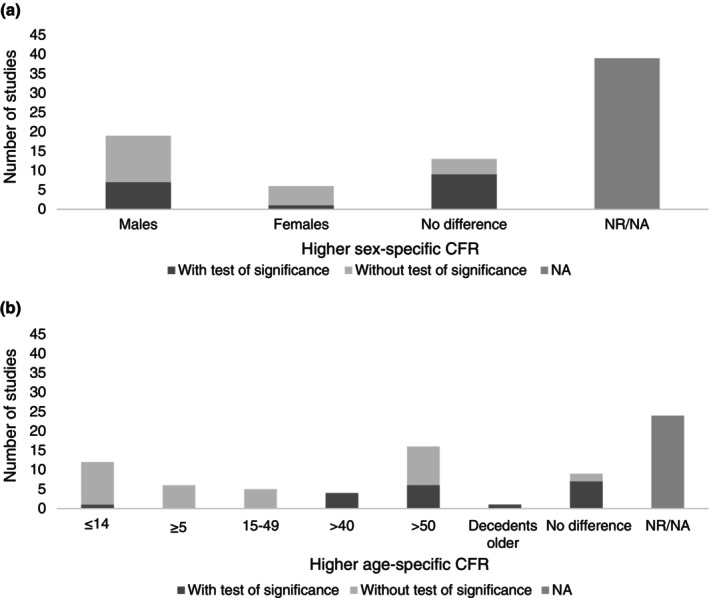
(a) Sex with the highest case fatality ratio (*n* = 77); (b) age groups with the highest case fatality ratio (*n* = 77).

Seventeen studies compared the sex‐specific CFRs (or the distribution of sex among decedents and survivors in case–control studies) using statistical models or tests (Figure 5a). Another 21 studies provided sex‐specific CFRs (or data to compute them) but they did not compare them with tests. In 19 studies, males had the highest CFR, and 13 found no difference comparing males and females. The CFR in males ranged from 0.6 [50] to 18.2 [47] and in females from 0.4 [57] to 16.1 [47]. More than half of the studies (39/77) reported neither sex‐specific CFR nor the data to compute it.

##### Age

The age‐specific CFR (or the age distribution of decedents and survivors in case–control studies) was extracted from 53 studies. There was heterogeneity in the definition of the age groups, and multiple studies presented the results in terms of continuous age. Collating and aggregating the findings was not straightforward, and there was age overlap in the categories (Figure 5b). Of the 53 studies, 12 found a statistical difference in CFR across the age groups, including four studies that reported higher CFR in those aged above 40 (i.e. >40, ≥45) and six above 50 (i.e. >50, >55, >60, >65). Seven studies did not find significant differences. The other 34 studies provided CFR values or data to derive CFRs but did not compare the different age groups with tests of significance. Overall, the CFR was higher among older age groups, especially those 50 or above, though 12 studies reported higher CFR among children less than 14 years (i.e. <1, <4, <5, <10, 5–14). Studies showed a range of CFRs between and within different age groups. For example, there were outbreaks with a lower CFR in the higher age group categories, that is, 0.4% among those ≥45 [78] while others reported CFRs as high as 20.0% (>60 years) [75]. Twenty‐four studies reported neither age‐specific CFR nor the data to compute it.

##### Comorbidities

References and analyses of comorbidities and cholera deaths were identified in five studies [22, 26, 55, 59, 67]. Lack of data and low power restricted the ability of the investigators to examine the association of cholera mortality with malaria and with chronic medical conditions such as cancer, tuberculosis or HIV [22, 59, 67]. One study found that the nutritional status of children less than 10 years was associated with increased mortality [45]. There were limited records referring to pregnancy: Ayangade commented that pregnant women are less likely to die because of *Vibrio cholerae* infection compared to non‐pregnant women [13] and Cartwright et al. found that pregnancy was not a risk factor for cholera mortality [22].

#### Healthcare

##### Access to care

Access to medical care was one of the most common constraints related to mortality, with studies reporting: (i) limited access to proper care [9, 44, 62]; (ii) access challenges due to remote or distant areas or areas only accessible by foot or inaccessible [12, 19, 27, 32, 49, 55, 60, 63, 65, 70]; (iii) transport problems [65, 76]; and (iv) health care, including CTCs, not available early in the outbreak [23, 77]. Tesfay and Biru partly attributed the acceptable CFR observed in three consecutive waves of cholera in Ethiopia to the decentralisation of cholera treatment units [78].

##### Case management

The thematic analysis yielded comments related to case management, such as delays in treatment at the facility [52], lack of fluid output monitoring [28, 40], premature discharge from facilities [53, 55] and overall inadequate and poor management [12, 42, 53, 55, 58, 59]. The authors described hydration issues such as inadequate initial hydration and delays in hydration [16, 28, 40, 69, 76], under‐utilisation of ORS [40, 76], not receiving hydration therapy or receiving IV alone [77], IV fluids not given to all patients [59] and over‐hydration [40]. Studies showed that cholera‐related deaths were negatively associated with early provision of ORS [22, 56], use of IV [22], provision of antibiotics [22], spending an additional night at the CTC [71], and hospitalisation [16, 35, 59].

##### Facility

Some of the factors related to cholera deaths included a shortage of supplies [55, 59, 65, 76], a lack of emergency resuscitation facilities [42], poor coordination between primary and secondary care [48], increased patient load, overcrowded facilities and long queues [63, 65], and a lack of supervision [55]. Shortages and a lack of skilled health workers [12, 41, 58, 59, 65, 76], a lack of knowledge among health workers [55] and a lack of experience in establishing intravenous infusions [40] were also perceived to contribute to cholera mortality. Al Abbassi et al. acknowledged the benefit of training healthcare workers on the correct use of rehydration fluids [8]. The availability of fluids for rehydration and ensuring the availability of supplies [8, 78] contributed to a lower CFR.

#### Health‐seeking behaviour

Cholera deaths were also attributed to delays in seeking care [8, 12, 28, 35, 42, 48, 49, 53, 63, 69, 79, 82], not seeking care at all [12, 22, 29, 35, 40, 55, 60, 62, 77], showing reluctance to visit government health facilities [63] and visiting village practitioners or “quack doctors” or low‐cost services [63, 70]. On the other hand, seeking care at cholera treatment centres, secondary hospitals, physicians, governmental facilities [35, 54, 59, 70], and presenting early [21, 67] were protective factors.

While three studies found that the home use of ORS did not have a protective effect [46, 56, 62], another study showed that patients who received rehydration at home had a lower odds of death [54]. Mortality studies reported that decedents lacked knowledge of ORS [53, 65], and did not use ORS at all or consumed inadequate amounts of ORS [53, 62, 65]. In addition to the ORS, Quick et al. (1993) also argued that home‐prepared rehydration solution did not have a protective effect, explaining, however, that the majority of the respondents did not know the correct recipe [62].

References to vaccination were limited. Two studies considered the vaccination status and found that most of the decedents were unvaccinated [16, 83] but did not have enough power [16]. Mutale et al. reported a non‐significant effect of vaccination [56].

### Time from symptom onset to seeking care, symptom onset to death and admission to death

The time to seek care at a health facility was documented in 12 papers. The time used varied and included periods of less than: 2 h [65]; 4 h [12] 6 h [53, 56]; 8 h [46]; 1 day [16, 21, 36]; 2 days [35]; and 5 days [67]. Other studies used time as a continuous variable and reported mean or median time from the onset of symptoms to admission [40, 48], of which one reported a longer duration for non‐survivors compared to survivors [48]. Elimian et al. showed that a delay of more than 2 days in seeking care doubled the odds of cholera‐related death [35] and Baltazar et al. stated that consultation within 5 days was protective against death [67].

According to the charted information, the majority of patients died within a period of 1–3 days from symptom onset [37, 53, 62, 77]. A longer median duration of symptoms prior to death was observed in facility decedents compared to community decedents [65]. Five studies reported that the majority of facility deaths occurred within the first 24 h of hospitalisation [37, 40, 59, 69, 76, 81]. Two studies reported 20.2% [28] and 75% [10] of deaths occurring within 4 h of arrival at the facility, respectively.

### Mortality assessment and studies that examined mortality risk factors

The final step of the analysis was the exploration of studies that specifically focussed on cholera mortality. A third of the included studies (*n* = 27/77) aimed to assess cholera mortality, identify reasons for lower or excess mortality, or determine risk and protective factors.

Of the 27 studies, 22 were analytical and applied significance tests or regression analysis to determine factors associated with mortality. The main risk factors identified in these studies are presented in Table 2. Moreover, the table lists other factors that were included in the analysis of each study but did not reach statistical significance. The number of reported decedents (decedents‐cases for case–control studies) ranged from 7 to 817. There was diversity in the factors examined across the different studies. Furthermore, variables found to be determinants of cholera mortality in one study were not significant for other studies.

**TABLE 2:** Analytical studies that examined cholera mortality—main characteristics and findings (*n* = 22).

Study	Country and period	Study design	Participants	Number of cholera cases	Number of fatal cases	Number of cases (fatal cases) versus controls (survivors)^a^	Statistical methods	Risk factors	Factors found not to be significant
Islam and Sahid [45]	Bangladesh 1980–1981	Secondary analysis	Patients admitted with diarrhoea	222	11	NA	Chi‐square tests	Nutritional status in children < 10 years	Age
Siddique et al. [70]	Bangladesh 1985	Outbreak investigation incl. case–control	Reported fatal cases and survivors affected by the disease	795	51	39 versus 31	Chi‐square tests	Female Lower socioeconomic status Treated village practitioners (vs. qualified doctors) Longer distance from health facility (among those of higher socioeconomic status)	Age
Quick et al. [62]	Peru 1991	Case–control	Reported cholera‐like fatal cases and survivors of episodes of diarrhoea	222	30	29 versus 61	Univariate regression	Treated only at home	Use of ORS Use of homemade sugar‐salt solution
Jacoby et al. [46]	Peru 1991	Case–control	Cholera fatal cases and survivors treated at the hospital	NA	NA	42 versus 109	Univariate & multivariable regression	Severe dehydration Among those 65 or above, arriving after 8 h of disease onset	Home use of ORS Age
Gunnlaugsson et al. [40]	Guinea‐Bissau 1994	Outbreak investigation incl. case–control	Persons who had a cholera‐like illness (fatal cases and survivors)	1169	62	16 versus 32	Univariate & multivariable regression	*Overall* Male Age ≥ 45 Catchment area + Age 2–14 *Case–control* In poor health/intoxicated at illness onset	*Overall* Distance to health centre *Case–control* Not attending a health centre
Ryan et al. [17]	Bangladesh 1996	Secondary analysis	Inpatients with microbiologically confirmed cholera	19,100 incl. 887 admissions	33	NA	Univariate & multivariable regression	Bacteraemia Radiographic evidence of pneumonia Acidosis	NA
Manga et al. [52]	Senegal 2004–2006	Secondary analysis	Reported cases and cases admitted to infectious diseases clinics	2942	30	NA	NA	Delay in treatment Age>60 Severe dehydration at admission	NA
Shikanga et al. [59]	Kenya 2008	Outbreak investigation incl. case–control	Reported cholera‐like illness cases and cases from active case finding	396	45	31 versus 55	Univariate & multivariable regression	+ Home antibiotic treatment + Hospitalisation + Treatment in government operated health facilities + Receiving education about cholera by health workers	*Univariate* Sex, educational level, having other cholera cases in the home, household crowding, duration of transport, transport fare to the nearest admitting facility, socioeconomic status, clinical presentation. *Multivariable* Protected water source for drinking water, safe stool disposal, stored water in narrow‐mouthed container, chlorine absent in home water, chronic medical condition (cancer, TB, HIV), not working at time of illness onset
Cartwright et al. [22]	Cameroon 2009	Cross‐sectional and case–control	Reported cases (fatal and survivors)	NA	NA	25 versus 72	Univariate regression	Age>50 Thatched roof Positive chlorine residual in stored water + Seek care outside home (any type of care, visited healthcare facility) + Received oral rehydration salts + Received intravenous fluids + Received antibiotics	Sex, religion, marital status, education, literacy, employment, household assets, water sources, water storage or water treatment practices, quantity of diarrhoea, symptoms, clinical comorbidities (pregnancy, alcohol use), treatments undertaken at home, transport time ≤ 20 min
Kolo et al. [48]	Nigeria 2011	Secondary analysis	Admitted cholera cases	1220	38	NA	T‐tests and Chi‐square tests	Age (decedents older) Longer duration of hospitalisation Longer duration of symptoms before hospitalisation Male	NA
Morof et al. [54]	Zimbabwe 2008–2009	Descriptive & Case–control	Community cases (died outside institution and survivors)	NA	NA	55 versus 110	Univariate & multivariable regression	Male + Received home‐based rehydration + Went to cholera treatment centre Among participants that did not go to CTC, married had lower odds of death (sensitivity analysis)	*Univariate* Married, religion, education, received information on cholera, access of ORS in village, could afford sugar *Multivariable* Average number of persons sleeping in the house at night, any income
Valcin et al. [81]	Haiti 2010–2011	Secondary analysis	Patients admitted to CTC	4070	15	NA	Univariate regression	Severe dehydration	NA
Page et al. [60]	Haiti 2011	Cross‐sectional	Rural communities (episodes of diarrheal illness and deaths)	2034	224	NA	Multivariable regression	Age ≥ 60 Greater severity of illness Living in remote areas (mode of transport foot) Not seeking care	District, sex
Bekolo et al. [16]	South Sudan 2014	Secondary analysis	Cholera cases seen at cholera treatment facilities	4115	62	NA	Univariate & multivariable regression	Severe disease Age ≥ 50 + Hospitalisation	Vaccination (no power to detect any association)
Djouma et al. [12]	Cameroon 2009–2011	Case–control	Community members who developed a cholera‐like syndrome (active case finding)	NA	NA	97 versus 187	Univariate & multivariable regression	Age <5 (in univariate, data not shown for multivariable) Household case management Management in a community temporary cholera treatment centre ≥4 h between onset of symptoms and the decision to seek care	*Univariate* Sex, year, taking medicine at home
Bwire et al. [19]	Uganda 2011–2015	Secondary analysis and cross‐sectional survey	Reported cases from outbreaks in fishing villages	1827	43	NA	Univariate & multivariable regression	Male Month of onset (July or September)	NA
Semá Baltazar et al. [67]	Mozambique 2011–2015	Secondary analysis	Reported suspected cases	1863	23	NA	Multivariable regression	Male Rice water stools Abdominal pain Leg cramps + Duration of 1–4 days between onset and consultation	Age, hospitalisation, attended a market in the last 7 days, primary source of drinking water
Sinyange et al. [71]	Zambia 2017–2018	Case–control	Cholera deaths and survivors	NA	NA	32 versus 64	Regression	+Additional night at a CTC^b^	Not reported
Hemmer et al. [41]	Cameroon 2004	Secondary analysis	Cholera cases in treatment centres	4915	63	NA	Chi‐square tests, univariate & multivariable regression	Age>40 0.5 (vs. <0.5) nurses per treatment place	Sex
Elimian et al. [35]	Nigeria 2018	Secondary analysis	Reported cases	41,394	815	NA	Univariate & multivariable regression	Age 41–59, ≥60 Male Peri‐urban setting Rainy season Flooding in 2018 >2 days to seek health Did not seek care (home) + Urban versus rural + Sought care at secondary hospital + Sought care at cholera treatment centre + Hospitalisation	*Univariate* Sample collected for rapid diagnostic test Positive rapid test outcome *Multivariable* Area under armed conflict
Mutale et al. [56]	Zambia 2018	Case–control	Reported cases: fatal (CTC or community) and patients admitted to CTC who were discharged alive	NA	NA	38 versus 76	Univariate regression	Age > 55 Education <primary + Immediately receiving oral rehydration solution	Sex, employment, household size, household assets, primary water source, household member with cholera, household shares latrine, cholera vaccination Care at home (ORS, time from illness to initiation of ORS, received antibiotics) Clinical symptoms, clinical care (received IV fluids, antibiotics, time from illness to arrival, duration of stay in CTC). Knowledge and behaviours of cholera and drinking water treatment
Bragança et al. [18]	Nigeria 2018	Secondary analysis	Patients admitted to CTC	500 children	7	NA	Fisher's tests	NA	Nutritional status Treated for dehydration Treatments given

Abbreviations: CTC, cholera treatment centre; IV, intravenous; ORS, oral rehydration solution; +, protective factor OR lower CFR.

^a^ For case–control studies.

^b^ Preliminary findings.

The other five studies [28, 37, 53, 55, 65] described decedents both in the community and in health facilities, without applying analytical methods (Table 3).

**TABLE 3:** Descriptive studies that examined cholera mortality—main characteristics and findings (*n* = 5).

Study	Country and period	Study design	Participants	Number of cases	Number of fatal cases	Description of fatal cases
Faruque and Eusof [37]	Bangladesh 1983	Cross‐sectional	Reported cases	NA	92	37% died within 12 h and 30% within 13–24 h 51% received care from village practitioner and 20% from qualified doctor 47% received IV therapy, ORS and antibiotics and 32% ORS alone
Routh et al. [65]	Haiti 2010	Cross‐sectional	Hospital and community fatal cases	NA	87	Facility versus community: More used ORS before seeking care Longer median time from illness onset to death Fewer reported receiving information about cholera after the outbreak started
Msyamboza et al. [55]	Malawi 1998–2012	Secondary analysis and cross‐sectional	Reported fatal cases	1806	38	47.4% died because of poor case management 26.3% were community deaths—did not seek care
Davies‐Teye et al. [28]	Ghana 2014	Secondary analysis	CTC fatal cases	20,199	121	Mean age 41 65.7% males 90.9% no health insurance 51% severe dehydration
McCrickard et al. [53]	Tanzania 2015–2016	Case‐series	Suspected cholera fatal cases through active case finding	NA	101	Median age 23 (2–80 years old) 57% male 59% community death 80% died within 24 h of symptom onset 10% consumed ORS

Abbreviations: CTC, cholera treatment centre; IV, intravenous; ORS, oral rehydration solution.

### Key recommendations

While undertaking the scoping review, we identified gaps in research and public health practices. Based on the information provided in the published studies, the following improvements to outbreak response and adaptations to clinical guidance are recommended (Box 1).

BOX 1 **Recommendations for analyses of outbreaks and further research.**

**Outbreak description (intra outbreak and post outbreak) (based on routinely‐collected data):**

‐ Analysis of the number of cases and CFR by age groups.
‐ Analysis of the number of cases and CFR by sex.
‐ Clear guidance on reporting of the number of deaths and CFR by location (facility vs. community) and analysis carried out accordingly.

**Research gaps:**

‐ Age and/or sex have been identified as risk factors for cholera mortality in some settings but there are limited explanations or hypotheses to explain the results. Further research is needed to explore and understand the patterns observed and data from outbreaks should be explored further. A systematic review and meta‐analysis would be useful to compile the evidence and understand which sex and age‐groups are most at risk.
‐ There are no comparisons in terms of the main characteristics of the patients per death location, that is, in facility and community. Such analysis is essential to explore who is not reaching care and why (access to care vs. health‐seeking behaviour, etc.).
‐ There are only a few reports on comorbidities and cholera mortality and analyses are often underpowered (due to the number of cases and deaths). Collecting and analysing information on comorbidities, co‐medications, clinical and laboratory results is important in understanding which underlying conditions could increase the risk of cholera deaths.

## DISCUSSION

This work presents a summary of factors contributing to cholera deaths, as provided in the 77 manuscripts included in the review. The identified reasons were context‐specific, and often interlinked, as cholera is a multi‐dimensional public health problem. Despite cholera being a disease of global importance for more than two centuries, our study highlights that there are still gaps in our understanding of potential risk factors for cholera mortality and that basic descriptive epidemiology that could help orient improvements in access to care for and clinical management of cholera patients is lacking.

The most frequently reported risk factors were old and young age and sex. However, not all the included studies reported these characteristics in their description or analysis. In many studies, the CFR or the distribution of age and sex groups among deaths was not directly provided. Age and sex are key demographic factors, and these data are routinely collected, both for outbreaks and surveillance purposes. The lack of published analysis suggests that collected data are not routinely analysed in the field. Analysing CFR per key demographic characteristic during epidemics is essential to orient community engagement, identify contextual barriers to accessing treatment, and to adapt response strategies. Available data from outbreaks could be analysed to explore further the factors related to cholera mortality. At the same time, a meta‐analysis would be useful to compile the evidence and understand which sex and age groups are most at risk.

Notably, in some contexts, deaths were more frequent among affected males than females; however, few papers included any explanation for this variation in CFR, and none described any field investigations carried out in order to identify and address potential barriers to accessing care or coexisting medical conditions or illnesses that might contribute to the likelihood of dying from severe cholera. Possible explanations included differences in health‐seeking behaviour between men and women, poor access to care due to work in remote areas [19] or lack of awareness of cholera in males [63]. Failure or delay to seek care may also be linked to socio‐economic reasons in settings where the man is the head and the provider of the family [63]. As factors limiting access are context‐specific, local operational research both prior to and during outbreaks is needed to compare the health‐seeking behaviour, knowledge and attitude about cholera and cholera mortality in females and males to engage communities and identify ways to reduce barriers to care.

Disentangling the effect of age and identifying the most vulnerable group was challenging, as there was a wide variation in the reporting of age. Different studies used different age group categories or reported age as a continuous variable. The reported data suggested that in many settings the CFR was higher among the elderly, especially those 50 years or above. Older patients may not have an adequate immune response towards the infectious agent and thus experience more severe disease [48] or may present late or never to treatment facilities. Two studies from Kenya commented that the elderly are often neglected as they rely on others for care [31, 75] and others suggested that the high CFR observed among those aged 65 or above could be associated with medical factors including comorbidities in this age group [25, 48]. While older age and associated comorbidities in older age groups could be a risk factor for cholera deaths, the absolute number of cases and deaths in the older age groups is likely to be a fraction of cases and deaths in the younger age groups. Thus, the effect of age should be explored further, taking also into consideration that age pyramids are changing in cholera‐affected countries, and the proportion of older people in any population is increasing.

Apart from the few aforementioned comments about older age and comorbidities, the evidence around comorbidities and cholera mortality was particularly scarce. It was suggested that in some settings, a higher CFR could be attributed to HIV and malaria [55]; nonetheless, the lack of data on underlying conditions did not permit testing of this hypothesis [67]. Only one of the included studies examined the association of chronic medical conditions (cancer, tuberculosis and HIV) and cholera mortality and was itself underpowered [59].

Collecting and analysing information on predisposing conditions could be time and resource consuming, especially during epidemics. Nevertheless, this information is crucial to understand who is at higher risk of death, and some simple tests including for malaria and glucose can be conducted in most settings, and access to a basic laboratory would permit further investigations. Therefore, a more systematic collection and analysis of data on underlying diseases is needed to adapt management recommendations.

The extracted information indicates that a considerable proportion of deaths still occurs in the community. Nevertheless, the true burden of cholera mortality both in the community and facility remains unknown. During a declared outbreak, diagnostic testing of all suspected cholera cases is not carried out, and patients with other infections may be counted as cholera cases and deaths. On the other hand, cholera deaths in the community are not always reported to the corresponding authorities. In 2024, the GTFCC published guidance on separate reporting of community and facility deaths and deaths on arrival, as well as on calculating the respective CFR [85, 86].

Limited or no access to care, delay in seeking care, or failure to do so may result in death in the community or en route to the facility. Late presentation may also contribute to facility deaths soon after arrival. Access to care is often impeded by the remoteness of the affected population [12, 38, 60, 66], insecurity in the area [16, 35] or following displacement as a result of flooding [26] or conflicts. Communities must be regularly engaged in order to ensure sustained provision of ORS for all patients with diarrhoea, and therefore also for cholera patients. Averting severe dehydration can help ensure patients arrive at treatment centres and increase the odds of survival both in the community and at a facility. At the same time, this can only be achieved if health‐seeking behaviour is improved. Health promotion activities in the community are essential to raising awareness about using ORS, home‐prepared rehydration solutions and seeking care early.

Poor case management of cholera patients at health facilities or temporary community treatment centres could increase the risk of death from cholera. Inadequate initial hydration, under‐utilisation of ORS or IV fluids, overhydration, and lack of monitoring fluid output were some of the key issues raised in the included studies. Community deaths were observed among patients that had been discharged from health facilities [53, 65], suggesting that patients were prematurely discharged and/or did not receive adequate instructions on signs to return to the treatment structure [55]. For facility deaths, the studies underlined constraints such as shortages of supplies, lack of knowledge and skills among healthcare workers or semi‐trained community workers, as well as a lack of supervision provided by trained medical and nursing personnel. Improving cholera case management and ensuring that the health facilities have enough supplies and trained personnel and that community health workers are also trained and have adequate supplies such as ORS can reduce cholera deaths.

### Limitations

The scoping review is subject to limitations. This work followed the scoping review methodology design, summarising the existing research without performing a quality appraisal of the studies. Moreover, a quantitative synthesis could not be undertaken due to the heterogeneity of the studies. Bias could have been introduced when selecting the databases for the search. Despite these limitations, the review permitted a comprehensive exploration and presentation of existing evidence about cholera mortality and identification of gaps without restrictions by the design of the included studies nor by publication bias.

## CONCLUSIONS

The current scoping review highlighted that there has been limited published evidence about factors that increase the risk of cholera‐related death. Collecting, reporting and analysing characteristics such as age, sex and underlying conditions can improve our understanding of cholera mortality risk factors and can guide future case management recommendations. Data from outbreaks could be analysed to explore further the factors related to cholera mortality. For an old disease, there is still much to learn and improve.

## AUTHOR CONTRIBUTIONS

DO and IC conceptualised the research idea. DP, KA, DO and IC participated in the investigation. DP conducted the analysis and prepared the original draft. KA, DO, IC and PB critically reviewed and edited the manuscript. KA, DO and IC supervised the project.

## FUNDING INFORMATION

Funding for this work was provided by the Bill and Melinda Gates Foundation. The funders did not participate or have input in the scoping review; they did not play any role in the study design, data collection and analysis, or preparation of the manuscript.

## CONFLICT OF INTEREST STATEMENT

The authors have no competing interests to declare.

## Supporting information


**TABLE S1:** PRISMA‐ScR statement checklist.

**TABLE S2:** Search strategy for each source and number of retrieved records.

**TABLE S3:** Thematic analysis of risk factors for cholera mortality.
