# Supplementary material for: Risk factors for cholera mortality: A scoping review
Source: Trop Med Int Health. 2025 Apr 2;30(5):332–50. doi: 10.1111/tmi.14106 (PMC12050166; doi:10.1111/tmi.14106)
Supplement: Supplementary file 2 — TABLE S2: Search strategy for each source and number of retrieved records. [file TMI-30-332-s001.docx]

**S2 Table.** Search strategy for each source and number of retrieved records.

| **Source and search strategy** | **Number of records** |
| --- | --- |
| **Pubmed**  (("cholera"[MeSH Terms] OR cholera[Text Word])) AND ("mortality"[MeSH Terms] OR mortality[Text Word] OR "death"[MeSH Terms] OR death[Text Word] OR "Fatal Outcome"[MeSH Terms] OR fatal[Text Word] OR "case fatality rate"[Text Word]) | 1,717 |
| **EMBASE (excluding MEDLINE)**  ('cholera'/exp OR 'cholera') AND ('mortality'/exp OR 'mortality' OR 'death'/exp OR 'death' OR 'fatality'/exp OR 'fatal' OR 'case fatality rate'/exp OR 'case fatality rate') AND ([embase]/lim OR [embase classic]/lim) | 2,547 |
| **Web of Science**  (ALL=(cholera)) AND (ALL=(Mortality) OR ALL=(death) OR ALL=(fatal) OR ALL=(case fatality rate)) | 2,005 |
| **LILACS/VHL (excluding MEDLINE)**  (mh:("Cholera") OR cholera) AND ((mh:("Mortality")) OR mortality OR ( mh:("Death")) OR death OR fatal OR "case fatality rate") AND ( db:("LILACS" OR "WHOLIS" OR "PAHOIRIS" OR "PAHO" OR "IBECS" OR "BINACIS" OR "HISA" OR "LIPECS" OR "CUMED" OR "LIS" OR "MedCarib" OR "MULTIMEDIA" OR "DECS" OR "DESASTRES" OR "SMS-SP" OR "ARGMSAL" OR "BDENF" OR "INDEXPSI" OR "MINSAPERU")) | 408 |
| **Scielo**  cholera AND (mortality OR death OR fatal OR "case fatality rate") | 37 |
| **African Journals Online**  cholera AND (mortality OR death OR fatal OR "case fatality rate") | 34 |
| **Cochrane**  (MeSH descriptor: [Cholera] explodes all trees OR cholera) AND (MeSH descriptor: [Mortality] explodes all trees OR mortality OR MeSH descriptor: [Death] explodes all trees OR death OR MeSH descriptor: [Fatal] explodes all trees OR fatal OR "case fatality rate") | 35 reviews  2 protocols  45 trials  4 clinical answers |
| **OpenGrey**  cholera AND (mortality OR death OR fatal OR "case fatality rate") | 5 |
