# Supplementary material for: Risk factors for cholera mortality: A scoping review
Source: Trop Med Int Health. 2025 Apr 2;30(5):332–50. doi: 10.1111/tmi.14106 (PMC12050166; doi:10.1111/tmi.14106)
Supplement: Supplementary file 3 — TABLE S3: Thematic analysis of risk factors for cholera mortality. [file TMI-30-332-s003.docx]

**S3 Table.** Thematic analysis of risk factors for cholera mortality

| **Patient** | **Risk Factor** |
| --- | --- |
| **Biological** | Sex [19,35,36,40,42,48,54,57,59,63,67,70,75] |
|  | Age [9,16,20,22,26,31,35,36,40–43,48,49,51,52,56,57,59,60,75,82,85] |
| **Health conditions** | Comorbidities / pre-existing illness [26,30,82] |
|  | Underlying infections (HIV, malaria) [55] |
|  | Concomitant infection (pneumonia/ pneumonia and spp. bacteraemia/ sepsis) [17] |
|  | Pneumopathy [73] |
|  | Heart disease [73] |
|  | Gestational hypertension [73] |
|  | Poor nutritional status (children <10) [45] |
|  | +Pregnancy[13] |
| **Healthcare** |  |
| **Access to care** | Limited access to proper care [9,44,62] |
|  | Areas only accessible by foot / hilly areas / long distance/ remote / inaccessible [12,19,27,32,49,55,60,63,65,70] |
|  | Transport problems [65,76] |
|  | Health care not available (early in the outbreak) [23,77] |
|  | + Decentralisation of cholera treatment unit [78] |
| **Case** | Delay in treatment at the facility [52] |
| **management** | Inadequate/poor management [12,42,53,55,58,59] vs good management [78] |
|  | Inadequate initial hydration/ delay in hydration [16,28,40,69,76] |
|  | Did not receive hydration therapy or received IV alone [77] vs + Immediate provision of ORS /received ORS [22,56] |
|  | Under-utilisation of ORS [40,76] |
|  | IV fluids not given to all patients [59] vs + Use of IV [22] |
|  | Lack of monitoring fluid output [28,40] |
|  | Over-hydration [40] |
|  | Premature discharge from facilities [53,55] |
|  | Relatively long duration between admission and death/longer hospitalisation [48,59] |
|  | + Received antibiotics [22] |
|  | + An additional night at the CTC [71] |
|  | + Hospitalisation [16,35,59] |
| **Facilities** | Shortage of supplies [55,59,65,76] |
|  | Lack of emergency resuscitation facilities [42] |
|  | Lack of knowledge among health workers [55] |
|  | Lack of experience in establishing intravenous infusions [40] |
|  | Semi-trained community-based health workers /untrained volunteers [12] |
|  | Lack of supervision [55] |
|  | Health workers shortages / lack of skilled health workers [12,41,58,59,65,76] |
|  | Patient load / overcrowded governmental health facilities / long queue [63,65] |
|  | Poor coordination between primary and secondary care [48] |
|  | + Availability of fluids for rehydration /ensuring availability of supplies [8,78] |
|  | + Training to use rehydration fluids [8] |
| **Health seeking behaviour** |  |
|  | Did not seek care / treated at home [12,22,29,35,40,55,60,62,77] vs + Went to a facility i.e. cholera treatment centre, secondary hospital, physicians , governmental facility [21,35,54,59,70] |
|  | Delay in seeking care / late presentation [8,12,28,35,42,48,49,53,63,69,79,82] vs Early presentation [21,67] |
|  | Went to temporary community treatment centre [12] |
|  | Visited village practitioners or “quack doctors” / used low-cost services [63,70] |
|  | Reluctance to visit government health facilities [63] |
|  | Did not consume ORS / inadequate dose [53,62,65] vs + Received home-based rehydration [54] |
|  | Lack of knowledge on ORS [53,65] |
|  | Did not think ORS would help [53] |
|  | Did not know the correct recipe for home-prepared rehydration solution [62] |
|  | + Vaccination [16,56,83] |
|  | + Home antibiotic treatment [59] |
|  |  |

IV : intravenous, ORS : oral rehydration solution, + : protective factor OR lower CFR
